# Cholera and Dysentery

**Published:** 1852-10

**Authors:** 


### DYSENTERY AND CHOLERA.

A sporadic case of dysentery is still occasionally seen in this city, but the disease has almost disappeared. In conversing with our medical friends, we find that the opiate practice is that which has given most satisfaction in this complaint during the past season. The opiate has been variously combined, as with sulphate of magnesia, turpentine, calomel, and astringents; but, in the practice of all, an opiate has entered into the treatment. Opium in some of its forms, has unquestionably been the efficient agent. In our judgment, next to opium, the saline purgatives have proved most beneficial. We have not seen calomel used with advantage in any case.

Cholera seems to linger in some parts of the country, but Louisville has happily remained exempt from the pestilence. During the last days of September, it was reported to be prevalent in Henderson, Ky., and to have carried off some of the best citizens of that place. The disease is now rarely heard of on boats plying the Mississippi or Ohio.

